# Meet the Insidious Players: Review of Viral Infections in Head and Neck Cancer Etiology with an Update on Clinical Trials

**DOI:** 10.3390/microorganisms9051001

**Published:** 2021-05-06

**Authors:** Lejla Mahmutović, Esma Bilajac, Altijana Hromić-Jahjefendić

**Affiliations:** Genetics and Bioengineering Department, Faculty of Engineering and Natural Sciences, International University of Sarajevo, 71000 Sarajevo, Bosnia and Herzegovina; lhalilovic@ius.edu.ba (L.M.); esma-hasic@hotmail.com (E.B.)

**Keywords:** head and neck cancer, human papillomavirus, Epstein-Barr virus, Hepatitis C, Hepatitis B, clinical trials

## Abstract

Head and neck cancers (HNC) occur in the upper aerodigestive tract and are among the most common cancers. The etiology of HNC is complex, involving many factors, including excessive tobacco and alcohol consumption; over the last two decades, oncogenic viruses have also been recognized as an important cause of HNC. Major etiological agents of nasopharynx carcinoma and oropharyngeal carcinoma include Epstein-Barr virus (EBV) and human papillomaviruses (HPVs), both of which are able to interfere with cell cycle control. Additionally, the association of hepatitis C and hepatitis B infection was observed in oral cavity, oropharyngeal, laryngeal, and nasopharyngeal cancers. Overall prognoses depend on anatomic site, stage, and viral status. Current treatment options, including radiotherapy, chemotherapy, targeted therapies and immunotherapies, are distributed in order to improve overall patient prognosis and survival rates. However, the interplay between viral genome sequences and the health, disease, geography, and ethnicity of the host are crucial for understanding the role of viruses and for development of potential personalized treatment and prevention strategies. This review provides the most comprehensive analysis to date of a vast field, including HNC risk factors, as well as viral mechanisms of infection and their role in HNC development. Additionally, currently available treatment options investigated through clinical practice are emphasized in the paper.

## 1. Introduction

**Figure 1:**
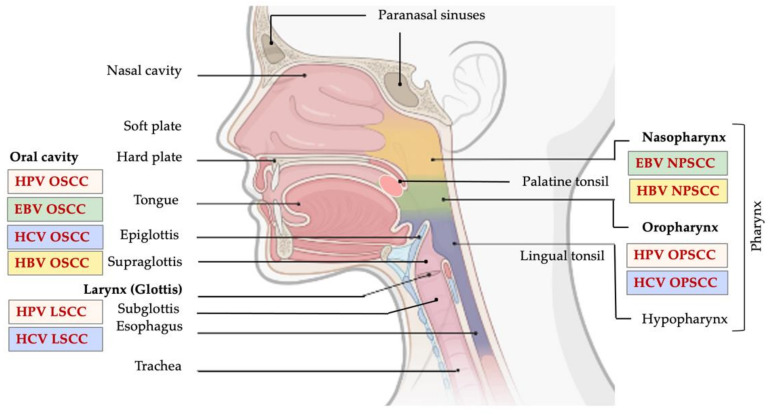
Anatomical model of head and neck region. The most common anatomical sites for HNSCC development include the nasopharynx, oropharynx, larynx, and oral cavity. The prevalence of each cancer type depends on the geographical area.

Head and neck cancer (HNC) comprises a heterogeneous group of upper aerodigestive tract neoplasms, and is among the most frequently diagnosed cancer type in the world [1]. According to a report from 2018, HNCs were identified as the eighth most common cancer, comprising 3% of all cancer diagnoses and 1.5% of cancer-related deaths in the U.S. [2,3]. Generally, the most common histologic type, accounting for more than 90% of all HNCs, is head and neck squamous cell carcinoma (HNSCC) [2,3,4]. HNSCC might arise in the mucosal surface lining of the upper aerodigestive tract, including: (i) oral squamous cell carcinomas (OSCC), arising from lips, buccal mucosa, hard palate, anterior tongue, floor of mouth, and retromolar trigone; (ii) oropharyngeal squamous cell carcinomas (OPSCC), which might arise from the base of the tongue, the soft palate, tonsils, uvula, and posterior pharyngeal wall; (iii) laryngeal squamous cell carcinomas (LSCC), arising from the supraglottis, glottis, subglottis; and (iv) nasal squamous cell carcinomas (NSCC), which develop from squamous epithelial cells lining the nasal cavity and paranasal sinuses (Figure 1) [4].

HNSCCs are very heterogeneous in nature and can vary histologically from well-differentiated, keratinized to undifferentiated, nonkeratinizing cancer types [4,5,6]. Around 10% of HNC can develop from lymphocytes, cells of connective tissue or of the salivary glands. The incidence and prevalence of different tumor types is usually influenced by the geographical position, population heterogeneity, and exposure to risk factors. 

Recent studies estimated that almost 85% of HNC risk is due to smoking or the consumption of other tobacco products, with 5–25 times higher risk for heavy smokers compared to nonsmokers [7]. Along with a reduced global incidence of smoking, rates of tobacco-related HNC have decreased most in North America and Western Europe [7]. However, this trend is not uniform across the world, and rates are still high in Eastern Europe and throughout Asia, mostly in China, India, and Indonesia [3,7]. In addition, increased alcohol consumption elevates the risk for HNC [2,3,7]. Consuming alcohol and tobacco might have a synergistic effect in causing HNC [8,9]. Additionally, chronic irritation, bad oral hygiene, occupational exposure, malnutrition, low fiber diets, and genetic factors have been associated with the development of oral cancer [3]. However, over the past decade, most diagnosed cases have arisen as a result of an infection with certain oncogenic viruses such as human papilloma virus (HPV), Epstein–Barr virus (EBV), Hepatitis C (HCV), or Hepatitis B (HBV) [3,5,10].

Treatment options for HNC are mainly dependent on the primary tumor site and tumor (T), node (N), and metastasis (M) TNM stage [11]. Patients diagnosed in early cancer stages usually undergo surgery and radiotherapy (RT), while patients in advanced and metastatic cancer stages undergo chemotherapy (CT), immunotherapy, or a combination of both to achieve more effective treatment responses [11,12,13]. Currently, surgery, RT, and CT represent standard care for HNC patients [14]. 

This paper aims to summarize current knowledge about the role of oncogenic viral infections, including HPV, EBV, HCV, and HBV in the pathogenesis of the HNC. Also, the currently available treatments and reports from clinical studies are summarized.

## 2. Human Papillomavirus

Papillomaviruses are among the oldest viruses known, dating back from the late Paleozoic era, i.e., 330 million years ago [15]. Over 90 million years ago, papillomaviruses with mucosal tropism started to develop, and during this long period, human papillomaviruses (HPVs), belonging to the *Papillomaviridae* family, evolved [16]. Almost 280 papillomavirus types have been detected in vertebrates, of which more than 200 types infect humans. HPVs have acquired the ability to utilize human cellular proteins to replicate and control the host cellular and immune systems at several levels in order to stay silent [15]. 

HPVs are widespread, making them a common risk factor for cancer development. They are classified into five genera, where Alphapapillomavirus, Betapapillomavirus, and Gammapapillomavirus are the most numerous. Clinically, most important mucosal HPVs belong to the alpha genus [15,16,17]. According to the World Health Organization International Agency for Research on Cancer (IARC) (Monograph Volume 100), HPVs are classified as carcinogenic to humans (Group 1), including HPV types 16 and 18; probably carcinogenic to humans (Group 2A) including types 31 and 33 and possibly carcinogenic to humans (Group 2B) including some types other than 16, 18, 31 and 33. The IARC has classified 12 HPV types (HPV-16, 18, 31, 33, 35, 39, 45, 51, 52, 56, 58 and 59) as carcinogenic to humans (Group 1) and high-risk (HR) types [18]. On the other hand, low risk (LR)-HPVs (6, 11, 40–44, 54, 61, 72, 81, etc.) are considered to be unrelated to carcinogenesis [6,16,18,19]. 

Even though the distribution of these most widespread HPVs varies in different geographic areas, HR-HPVs-16, 18, 31, 33 and 45 cause approximately 75% of all HPV-associated squamous cell carcinoma and 94% of all adenocarcinomas [15,20]. The HPV-16 and HPV-18 have the highest prevalence in HPV-positive OPSCC [21,22,23], whereas HPV-31, 33, and 35 are rarely implicated in OPSCC development [23]. LR mucosal HPVs, HPV-6 and HPV-11, generally cause benign papilloma/condyloma, whereas high-risk mucosal HPVs, such as HPV-16 and HPV-18, cause squamous intraepithelial lesions which can evolve to squamous cell carcinoma in the head and neck region and/or anogenital tract [15,20].

### 2.1. HPV Genome and Mechanism of Infection

HPVs are nonenveloped viruses with circular double-strand DNA of approximately 8000 bp in length (Table 1) [16]. The viral genome has three regions: (i) early (E) region including E1, E2, E4, E5, E6 and E7 oncogenes important in regulating viral DNA transcription and replication; (ii) late (L) region, encoding major (L1) and minor (L2) major and secondary capsid proteins; and (iii) long control region (LCR), a noncoding region positioned between the L1 and E6 open reading frames (ORFs) [16,18]. The LCR contains most of the regulatory elements involved in viral DNA replication and transcription, including the replication origin (ori) [16,18,24].

HPV infection arises through tissue scratches which provide the virus with accessibility to basal keratinocytes. The integration of HPV in host genome is a critical event in cancer progression. In early low-grade lesions, HPV genome is found in the episomal form, whereas integration of the viral genome occurs late in the pathogenesis of cancer [25,26]. The process of HPV genome integration into the host genome is still not completely understood. However, it is believed that DNA damage can facilitate the integration of the viral genome by generating breaks in both the viral and host genomes [26]. Integration breakpoints generally take place in the HPV E1 or E2 genes [25,26]. The E2 ORF is the preferential site of integration due to frequent disruptions and deletions compared to any other site. The E2 protein is a negative regulator of E6 and E7 expression, and loss of E2 ORF during integration enhances expression of the transforming E6 and E7 oncoproteins which are responsible for cellular transformation [26].

The viral oncoproteins E6 and E7 are essential for malignant conversion [24,27]. E6 induces transcription of human telomerase reverse transcriptase (hTERT) which elongates telomeres and prevents end-to-end fusion of chromosomes and apoptosis. Thus, the E6 oncoprotein has a fundamental role in cell immortalization. [15,24,27]. However, the most studied role of E6 oncoprotein is its ability to bind to p53 to form a ternary complex with E6AP ubiquitin ligase and p53 (E6-E6AP-p53), leading to p53 degradation via a proteasome-mediated pathway [15,16,24,27]. The E7 protein binds to the conserved motif of the retinoblastoma (Rb) protein, resulting in the transcriptional factor E2F nuclear translocation, which stimulates uncontrolled replication and cell-division [16,28].

Other cancer-related pathway proteins involved in the cell cycle regulation are also targeted by E6 and E7 oncoproteins. E7 proteins can inactivate cyclin-depend kinase (CDK) cell cycle inhibitors, including p21^CIP1^ and p27^Kip1^, and enhance activity of cyclin-dependent kinase 2 (CDK2) [29]. Moreover, E6 and E7 oncoproteins indirectly interfere with noncoding RNAs (microRNAs; miRNA) involved in tumor progression/suppression [24]. E6 and E7 increase oncogenic miRNA-21 levels which decreases expression of tumor suppressor PTEN gene. Based on many studies in HNC and cervical cancer, it was concluded that HPV oncogenes can enhance or decrease miRNA levels in HPV-associated cancer, and their effect depends on targeting either tumor suppressor or proto-oncogenes [15,16,24].

Expression of E6 and E7 oncoproteins can be regulated by epigenetic mechanisms, mostly by DNA methylation [30,31]. Integration of HPV DNA into the host genome during cancer progression causes structural disruption which prevents the transcription of genes positioned at 5’ of the long control region (LCR) and DNA methylation. Selective methylation in the viral LCR can cause overexpression of early viral proteins [32]. E2 is the main HPV regulatory protein which controls the transcription of HPV early proteins through a negative feedback control. LCRs in the mucosal HPV genome contain 4 E2 binding sites (E2BS) [33,34]. E2BS1 has the highest affinity for the E2 protein and is positioned towards the LCR 5’ end. Recruiting E2 to E2BS1 activates the viral early promoter, enhancing the transcription of HPV early proteins, including E6, E7, and E2 itself. Increasing E2 levels forces E2 proteins to bind to the E2BS3 and E2BS4 near the 3’ end of the viral LCR, which represses the early promoter and hinders transcription of early proteins [26,34,35]. However, CpG methylation in the E2BSs inhibits E2 binding, obstructing E2 regulation of viral early promoter. This event leads to overexpression of HPV E6 and E7 oncoproteins, and elevated odds of tumorigenesis [32].

Reports aiming to examine LCR methylation in HNC have described fluctuating CpG methylation levels, suggesting that methylation in LCR is not common to all HPV-driven HNC [31]. In cervical cancer, however, CpG methylation has also been observed in HPV L1 and L2 genes, whereas HPV DNA methylation in these sites has not yet been broadly investigated in HPV-driven HNC, even though current literature proposes similar methylation patterns in HNC [30,31,32]. Many studies have proposed DNA methylation as a biomarker to characterize malignant lesions [32].

The indicators of HPV infections can be numerous, from asymptomatic infections to benign warts or malignant lesions, intraepithelial neoplasia, and invasive carcinoma [15,16]. Generally, oral HPV infections are associated with sexual behavior, but recent evidence supports horizontal, mouth-to-mouth, virus transmission. Also, most HPV infections in newborns are transmitted vertically from the mother during pregnancy, delivery, or later, by saliva [15].

**Table 1:** Oncogenic viruses and their mode of action in HNC development.

Oncogenic Virus/Parameter		Human Papillomavirus (HPV)		Herpesvirus	Hepatitis Virus	
		HPV-Positive	HPV-Negative	Epstein-Barr Virus (EBV)	Hepatitis C	Hepatitis B
**Virus-related**	Nucleic acid	Circular double stranded DNA [16]	−	Linear double stranded DNA [36]	Single stranded RNA [37]	Double stranded circular DNA [38,39]
	Genome	Approximately 8 kb in size [16]	−	Approximately 180 kb in size [36]	9 600 bp in size [37,40]	The smallest genome with 3200 bp in size [38]
	Tropism	Kerationocytes and mucosal sufraces [16]	−	B-cells and epithelial cells [10,41]	Hepatocytes, lymphocytes, and salivary gland cells [42]	Hepatocytes and lymphocytes [43]
	Major viral oncoproteins	E6, E7 [15,16,24]	−	LMP1, LMP2A, EBNA1 [10,44]	NS3 or NS5A [40,42,45]	S, C, P and X [39,46]
	Virus transmission mode	Sexual contact, self-inoculation, vertical and horizontal transmissions [47]	−	Sexual contact, blood or saliva transmission [10]	Vertical transmission, horizontal transmission (sex or sharing of drug-injection needles) [39]	Sexual contact, self-inoculation, vertical and horizontal transmissions [47]
**Cancer-related**	Anatomic site	Oral cavity, oropharynx, larynx [47,48]	All sites, but mostly oropharynx [36]	Nasopharynx [49] Oral cavity [50]	Oral cavity, oropharynx, larynx [45,51,52,53,54]	Oral cavity [54] Nasopharynx [43,55]
	Histology	Nonkeratinized [56]	Keratinized [41]	Undifferentiated type NPC, squamous cell and non–keratinizing NPC [57]	Squamous cell [51,53]	Squamous cell or adenocarcinoma [58]
	Age	Under age of 50 [6]	Above age of 50 [6]	Above age of 50 [49,59]	Above age of 50 [42,54]	Above age of 50 [43]
	Gender	Mostly male [24]	Mostly male [24]	Mosty male [10]	Mostly male, 6.7-fold higher risk in male [42,54]	No significant difference [42,43]
	Incidence trend	Increasing	Decreasing	Increasing	Increasing	Increasing

### 2.2. HPV in HNSCC Development

The frequency of HPV-positive HNC is increasing drastically, specifically, that of oropharyngeal cancer (OPC). OPC has the highest incidence among HPV-positive cancers, with different distribution compared to HPV-negative OPC. Data have shown that more than 70% of oropharyngeal cancers are caused by HPV, and that HPV type 16 (HPV-16) causes almost 90% of the HPV-positive OPC in the United States [36]. 

Studies have shown that approximately 25% of HNC cases worldwide are caused by oral HPV transmission, mainly by oral sexual activities. However, this percentage differs between anatomical sites, i.e., between oral, oropharyngeal and laryngeal regions [47]. Increased number of oral sexual partners drastically increases oral transmission of HPV which ultimately leads to an escalation in infections in the head and neck region. Much higher number of HPV infections of the head and neck region is reported in men compared to women (Table 1) [47]. Globally, HPVs cause roughly 33.6% of nonkeratinized OPSCC, and 22.2% and 20.2% of OSCC and LSCC, respectively [56] (Figure 1). However, it is still completely unclear why the prevalence of HPV-positive HNC differs between anatomical sites [47]. 

HPV-positive OPC is considered entirely different than the traditional tobacco and alcohol-related OPCs, with a better prognosis compared to HPV-negative OPC [4]. It is important to understand that HPV status determines the criteria by which to analyze the cancer stage and provide an overall prognosis. Since pathology reports for OPC lacking HPV status can be misinterpreted, HPV testing is now suggested for all patients with newly diagnosed OPSCC [4]. Patients diagnosed with HPV-positive HNCs are younger, have more sexual partners and earlier ages of onset of sexual activity. Additionally, these patients are characterized with favorable health profiles and statistically low disease comorbidities than those observed in long-term smokers and heavy alcohol users [6]. Currently, there is a significant difference in HPV-positive and -negative OPC prognoses. However, this is not the same for HPV-positive and -negative oral cancers, and the role of HPV in oral cancers has not yet been fully elucidated [60].

Additionally, HPV-16 infection of the antral tissue of the nasal cavity has been suggested to be involved in the onset of Killian polyps (KP) [61]. KP is a benign lesion arising from the maxillary sinus region with still unknown etiology. Between 2000 and 2014, only a few studies were published on HPV association with nasal polyps, with highly inconsistent results, i.e., HPV-DNA prevalences ranging from 0% to 40% [61]. These discrepancies may be attributable to factors such as race, geographical area, laboratory procedures, tissue fixation, number of samples, and choice of controls [61].

However, there is no evidence claiming neoplastic transformation of KP, except reported incidents mimicking malignant transformation [62]. It is difficult to report HPV carcinogenesis in KP, as patients remove them immediately after presentation, and carcinogenic events caused by HPV infection require longer to develop and to be detected [63]. A study by Knör and colleagues indicated the existence of HPV in nasal and antrochoanal polyps. However, the association did not reflect any oncogene transformation [61]. Similarly, Oton-Gonzalez et al. (2021) reported the presence of low HPV-16 DNA amounts in both episomal and integrated forms in antral KP. However, they did not report viral expression of E2, E5, E6, and E7 sequences in the HPV-positive KP samples [63].

Detecting HPV DNA only is not adequate to define tumor causality. Thus, the expression of several markers is required to distinguish between HPV and non-HPV-related cases of head and neck carcinoma [19]. It has been suggested that tumor suppressor genes p16^INK4a^ and E6/E7 mRNA are sensitive biomarkers in detecting HPV-positive OPSCC in comparison to OSCC and LSCC. The weak sensitivity in detecting HPV-positive OSCC and HPV-positive LSCC contributes to the discrepancy in the prevalence of HPV-positive HNC between the three anatomical regions [47].

### 2.3. Current Treatment Options and Update on Clinical Trials

Treatment approaches for HNC subtypes, specifically OPSCC, are also dependent on HPV status, with more favorable outcomes and overall survival (OS) rates for HPV-positive patients [64,65,66]. Accordingly, clinical studies suggest a three-year OS of 82.4% and 57.1% in HPV-positive compared to HPV-negative patients, respectively [67,68]. Although the molecular profiles of the two groups of patients are different, treatment strategies for HNC patients usually follow the same therapeutic regimens, suggesting a need for novel treatment approaches that reflect the molecular background of HPV-positive and -negative patients [68].

Currently, RT is one of the most frequently used treatments for HNSCC patients in early and advanced disease stages, with 50% OS rate in early diagnosed cases [69] and 10–25% OS in advanced stages [70]. Interestingly, HPV-positive HNSCC patients are found to be more sensitive to RT, possibly due to the hypoxic status where low oxygen levels reduce therapeutic efficacy [68]. 

In past decades, surgical procedures of HNC have been challenging due to the complex anatomical structures and the inability to reach distal organ portions, resulting in surgery complications [71]. Advances in surgical technology have led to two approaches, i.e., transoral laser microsurgery (TLM) and transoral robotic surgery (TORS), with clinically significant outcomes [72,73]. Compared to TLM, TORS uses the Da Vinci Robot, allowing more precise tissue dissection that minimizes surgical morbidity. Considering safety, flexibility, and improved functional outcome of the patients compared to other surgical procedures, TORS was approved by the Food and Drug Administration (FDA) in 2009 for transoral resections of head and neck tumor lesions, with major application in patients diagnosed with OPSCC [72,74]. Recently, surgical procedures involving TLM or TORS are also preferred for oral cavity SCC, as well as for advanced stages of laryngeal and hypopharyngeal cancer [75,76]. Despite promising clinical outcomes of patients after TORS, however, RT and CT cannot be avoided in advanced disease stages [77].

Several clinical trials for TORS have been registered, mainly regarding to OPSCC. Moore et al. (2018) evaluated TORS outcomes for OPSCC patients (*n* = 314) ± adjuvant RT or CT [78]. From the total number of patients with known HPV status (*n* = 309), 93% (*n* = 286) were HPV-positive OPSCC. In this study, 24% of patients were treated with surgery alone, while postsurgical RT and CRT were applied to 28% and 48%, respectively. Five years after the surgical procedure, the study results indicated recurrence-free survival (RFS), distant-metastasis-free survival (DMFS), OS, and cancer-specific survival (CSS) rates of 92%, 90%, 86%, and 94%, respectively (Table 2). The five-year recurrence rate of the patients enrolled in this study was 8%, with increased risk in the group that underwent surgery alone [78]. Additionally, it is important to consider that this procedure might cause serious complications, including postoperative hemorrhage in 3–8% of the patients [74,79]. Even though HPV-16-positive patients have high post-therapeutic survival rates, still certain factors such as swallowing function after surgery and long-term toxicity significantly affect patient quality of life (QoL). An ongoing clinical trial (NCT02215265, PATHOS) with an estimated enrollment of 1100 patients is evaluating whether reduction of RT intensity in intermediate-risk OPSCC patients or CT dispense in higher-risk patients will cause favorable swallowing function after minimally invasive surgery. The estimated completion time of the study is 2027 [80].

According to recent findings, 80% of the patients who underwent TORS received RT which, at the same time, improved OS rates. Importantly, the radiation dose is usually low (60–66 Gy vs. 70 Gy) in cases receiving TORS as the initial treatment [81]. A number of clinical trials evaluating TORS following RT and CRT are currently ongoing (Table 2) [82]. In a randomized, phase II ECOG-ACRIN 3311 clinical trial, Ferris et al. (2020) evaluated the effects of low dose RT in HPV-positive, stage III-IVA OPSCC patients (*n* = 511) after TORS procedure. Patients were divided into four experimental groups (Arm A–D) (Table 2). The results indicated two-year progression free survival (PFS) > 90% in all study groups (detailed results in Table 2). Former data suggest favorable oncogenic outcome of TORS in p16-positive OPSCC patients with intermediate recurrence risk, while low-risk disease patients do not require postsurgical therapeutic interventions [77]. 

With the aim of decreasing high RT toxicity rates of nearby tissues and improving clinical outcomes, different approaches for HNC treatment have been recently applied, either as single treatment or in combination with other therapies, including several chemotherapeutic agents [70]. So far, FDA approved CT agents for the treatment of HNC include platinum-based therapy cisplatin and carboplatin, as well as 5-fluorouracil (5-FU), bleomycin, docetaxel and methotrexate with response rates up to 40% [14]. The results of meta-analyses and a number of clinical trials have suggested that the use of combinatorial therapy containing cisplatin, 5-FU, and docetaxel (TPF regimen) improves clinical outcomes with acceptable toxic rates among HNSCC patients [14,66].

**Table 2:** Clinical trials in HPV-related head and neck cancers.

Clinical Trial/NCT Number/Year	Phase	Disease Stage	Patient Number (*n*)	Treatment Arms	Outcome
2007–2015 [78]	N/A	OPSCC, stage I-IVb	*n* = 314 (*n* = 286 HPV-positive)	Arm 1: TORS^4^; Arm 2: TORS + RT^6^ (50–70 Gy); Arm 3: TORS + RT (30–70 Gy) + adjuvant CT (CDDP^1^/Carboplatin/Docetaxel/Cetuximab)	5-year after surgery LR^13^ RFS^17^: 92% DMFS^18^: 90% OS^8^: 86% CSS^3^: 94%
ORATOR NCT01590355 2012–2019 [82]	Phase II	Early stage OPSCC	*n* = 68 (*n* = 60 p16-positive)	Arm 1: RT ± CT^7^ (70/63/56 Gy in 35 fxs for 7 weeks); Arm 2: TORS + neck dissection	Median follow-up (27 months): Arm 1: 25 months Arm 2: 29 months QoL^11^ (MDADI score) Arm 1: 86.9 Arm 2: 80.1
ECOG-ACRIN 3311 NCT01898494 2013–2020 [77]	Phase II	HPV-positive, stage III-Iva OPSCC	*n* = 353	Arm A: TORS; Arm B: TORS, low-dose IMRT (50 Gy); Arm C: TORS, standard-dose IMRT (60 Gy); Arm D: TORS, standard-dose IMRT (60–66 Gy) + CT (weekly CDDP 40 mg/m^2^)	2-year PFS^12^: Arm A: 93.9% Arm B: 95.0% Arm C: 95.9% Arm D: 90.5%
ADEPT NCT01687413 2012–2020 [83]	Phase III	p16-positive OPSCC	*n* = 42	Experimental group: postoperative IMRT (60 Gy in 30 fxs); Active comparator: RT (60 Gy in 30 fxs) + cisplatin (40 mg/m^2^ × 6 doses)	1-year DFS: 100% vs. 90.9% 2-year LR control: 96.3% vs. 81.8% 2-year DM: 7.4% vs. 0%
ECOG-E1308 NCT01084083 2010–2015 [84]	Phase II	HPV-positive and/or p16-positive stage III-IV OPSCC	*n* = 80	Group 1: CDDP (75 mg/m^2^) and paclitaxel (90 mg/m^2^), low dose IMRT (54 Gy in 27 fxs × 5 weeks), cetuximab (400 mg/m^2^ → 250 mg/m^2^); Group 2: CDDP (75 mg/m^2^) and paclitaxel (90 mg/m^2^), standard dose IMRT (69.3 Gy in 33 fxs × 6 week), cetuximab (400 mg/m^2^ → 250 mg/m^2^)	2-year PFS and OS: 64% vs. 91% (IMRT 54 Gy); Primary CRR^19^: 73%
NCT01530997 2012–2020 [81]	Phase II	HPV-positive and/or p16-positive OPSCC, T0-T3, N0-N2c, M0	*n* = 43; HPV+/p16+: 63.6% HPV−/p16+: 36.4%	De-intensification chemoradiation therapy; IMRT (54–60 Gy) + CDDP (30 mg/m^2^ × 6 doses + limited surgical evaluation	pCR^20^: 86% 2-year LC^14^: 100%
NCT02281955 2014–2020 [81,85]	Phase II	HPV-positive and/or p16-positive OPSCC, T0-T3, N0-N2c, M0	*n* = 113; HPV+/p16+: 40.4 % HPV−/p16+: 10.5% HPV unknown/p16+: 49.1%	IMRT (60 Gy, 2Gy/fx) + CDDP (30–40 mg/m^2^ × 6 doses) or cetuximab (250 mg/m^2^) or Carboplatin (AUC 1.5 and paclitaxel 45 mg/m^2^) or Carboplatin AUC 3 + surgical evaluation	2-year outcome: PFS: 88.4% LC: 96.4% RC^15^: 98.2% LRC^16^: 94.6% DMFS: 92.0% OS: 93.0%
RTOG 0129 NCT00047008 2003–2014 [67]	Phase III	Stage III-IV SCC of oral cavity, oropharynx, hypohparynx, larynx, T2, N2-3, M0 or T3-4)	*n* = 721; HPV+ (*n* = 206), HPV− (*n* = 117)	Arm 1: Standard fractionation RT (70 Gy in 35 fx, 2 Gy/fx) + CDDP (100 mg/m^2^); Arm 2: Accelerated fractionation RT (72 Gy in 42 fx) + CDDP (100 mg/m^2^)	3-year outcome: OS (Arm 1 and Arm 2): 64.3% vs. 70.3%; OS (HPV-positive and HPV-negative group): 82.4% vs. 57.1%; PFS (HPV-positive and HPV-negative group): 73.7% vs. 43.4%
NCT01663259 2012–2020 (www.clinicaltrial.gov; accessed on 23 March 2021)	N/A	Stage III-IV (excluding N3 or T4), HPV-positive and/or p16-positive OPSCC	*n* = 42; HPV-positive (*n* = 42)	Cetuximab (400 mg/m^2^ → 250 mg/m^2^ concurrent with RT (70 Gy in 35 fx, 50–60 Gy)	2-year outcome: RR^21^: 19% DFS: 81% OS: 95.2% FFLRP^22^: 87.9%
CheckMate 141 NCT02105636 2014–2019 [86]	Phase III	Platinum-refractory, recurrent HNSCC	*n* = 361; Arm 1 (*n* = 240): p16-positive (*n* = 63) p16-negative (*n* = 50); Arm 2 (*n* = 121): p16+ (*n* = 29) p16− (*n* = 36)	Arm 1: Nivolumab (3 mg/kg, IV, every 2 weeks); Arm 2: Cetuximab/Methotrexate/Docetaxel (Cetuximab 400 mg/m^2^ → 250 mg/m^2^ or methotrexate 40–60 mg/m^2^ or docetaxel 30–40 mg/m^2^, weekly)	18-month OS (Arm 1 and Arm 2): 7.49 vs. 5.06 months; 1-year OS: 36.0% vs. 16.6%; 6-month PFS: 19.7% vs. 9.9%; RR: 13.3% vs. 5.8%; Median OS in p16-positive patients: 9.1 vs. 4.4 months; Median OS in p16-negative patients: 7.5 vs. 5.8 months
KEYNOTE-012 NCT01848834 2013–2020 [87]	Phase Ib	PD-L1-positive, R/M^5^ HNSCC	*n* = 60; HPV-positive (*n* = 23), HPV-negative (*n* = 37)	Pembrolizumab (10 mg/kg, once every 2 weeks)	OR^9^ (central vs. investigator review): 18% vs. 21%; OS: 13 months
KEYNOTE-040 NCT02252042 2014–2020 [88]	Phase III	R/M HNSCC	*n* = 495;HPV-positive (*n* = 119), HPV-negative (*n* = 376)	Pembrolizumab group (200 mg, 3-week cycle); Active comparator group (Methotrexate 40–60 mg/m^2^ or docetaxel 75 mg/m^2^ or cetuximab 400 mg/m^2^ → 250 mg/m^2^)	2-year outcome: OS (pembrolizumab vs. active comparator group): 8.4 vs. 6.9 months; PFS: 2.1 vs. 2.3 months ORR^10^: 14.6% vs. 10.1%; DOR ^23^: 18.4 vs. 5.0 months
KEYNOTE-048 NCT02358031 2015–2020 [89]	Phase III	R/M HNSCC	*n* = 882; HPV-positive (*n* = 190), HPV-negative (*n* = 692)	Pembrolizumab monotherapy (200 mg of 3-week cycle for 2 years); Pembrolizumab + CT (200 mg of 3-week cycle for 2 years + cisplatin 100 mg/m^2^ or carboplatin (AUC 5 + 5-FU ^2^ 1000 mg/m^2^ up to 6 cycles); Cetuximab + CT (Control) (400 mg/m^2^ → 250 mg/m^2^ + cisplatin 100 mg/m^2^ or carboplatin (AUC 5 + 5-FU 1000 mg/m^2^ up to 6 cycles)	47 months outcome: OS (Pembrolizumab + CT group vs. control group): 13.0 vs. 10.7 months; OS in PD-L1 CPS > 1 participants: 13.6 vs. 10.4 months; OS (Pembrolizumab monotherapy vs. control group): 11.5 vs. 10.7 months

CDDP^1^—cisplatin; 5-FU^2^—5-fluorouracil; CSS^3^—cancer-specific survival; TORS^4^—transoral robotic surgery; R/M^5^—recurrent or metastatic; RT^6^—radiotherapy; CT^7^—chemotherapy; OS^8^—overall survival; OR^9^—overall response; ORR^10^—objective response rate; QoL^11^—quality of life; PFS^12^—progression free survival; LR^13^—loco-regional; LC^14^—local control; RC^15^—regional control; LRC^16^—loco-regional control; RFS^17^—recurrence-free survival; DMFS^18^—distant metastasis-free survival; CRR^19^—clinical response rate; pCR^20^—pathologic complete response; RR ^21^—recurrence rate; FFLRP^22^—freedom from local regional progression; DOR^23^—duration of response.

Patients who fail to receive appropriate systemic agents or belong to the category not eligible of undergoing surgery or concurrent CT are usually treated with RT alone [70]. In a clinical study involving HPV-positive stage I–IV OPSCC, O’Sullivan et al. (2012) evaluated the outcome in patients treated with RT alone (*n* = 148 HPV+, *n* = 59 HPV−). The results have indicated increased three-year OS in HPV-positive patients (81%) versus HPV-negative patients (44%). Interestingly, OS in p16-positive patients with a less than 10-pack-year smoking history was similar in the RT-alone treatment and CT treatment groups (86% vs. 88%), respectively [90]. 

As mentioned previously, HPV-positive patients have better prognoses and OS rates compared to HPV-patients [67,68]. In accordance with this, treatment de-intensification strategies are considered in HNC patients with HPV-positive status in order to reduce long-term toxicity but also improve therapeutic outcomes [70]. In a phase III RTOG 0129 clinical trial, Ang and colleagues evaluated the association of OS rates and HPV status in stage III-IV OPSCC patients. Patients were treated with accelerated-fractionation RT and standard-fractionation RT, both in combination with cisplatin. The results indicated nonsignificant change in three-year OS between accelerated-fractionation RT and standard-fractionation RT, 70.3% vs. 64.3%, respectively. However, HPV-positive OPSCC patients (*n* = 206 of 323) had a better three-year OS (82.4%) compared to HPV-negative OPSCC patients (57.1%), as well as reduction in risk of death for 58% [67].

Moreover, a postoperative adjuvant de-intensification treatment strategy was evaluated in phase III clinical trial (ADEPT, NCT01687413) with HPV-related, p16-positive OPSCC patients (*n* = 42). The patients were divided into two treatment arms, i.e., IMRT or IMRT in combination with standard cisplatin dose (Table 2). The results indicated one-year DFS of 100% in IMRT group compared to IMRT in combination with cisplatin (90.9%). In addition, two-year loco-regional control (LRC) between the two treatment groups were 96.3% vs.81.8%, respectively. Although more favorable treatment outcomes in regard to abovementioned parameters were observed in patients receiving IMRT only, two-year distant metastasis did not occur in IMRT and cisplatin-treated group (0%), while 7.4% of the patients in IMRT group experienced distant metastasis [83].

An open-label, phase II study (E1308, NCT01084083) enrolling patients of stage III–IV HPV-positive OPSCC has evaluated the ability of reduction in radiation dose in patients with complete CR to induction CT. The patients received induction CT including cisplatin, paclitaxel and cetuximab, following IMRT (54 Gy and 69.3 Gy) with cetuximab. Patients characterized with primary-site CR and treated with lower IMRT have shown two-year PFS and OS of 80% and 94%, respectively. Additionally, after one-year follow up, patients receiving 54 Gy radiation dose had less swallowing difficulties (40% vs. 89%) [11]. Dose de-escalation of adjuvant RT aiming to lower long-term toxic rates has been evaluated in multiple clinical trials (NCT01560997, NCT02281955) [91], as summarized in Table 2.

Despite the treatment advances in HNSCC patients implicating surgery, RT, and CT, the prognosis still remains poor, especially in recurrent/metastatic HNSCC, regardless of HPV status [92]. Consequently, cetuximab, an IgG1-subclass anti-EGFR monoclonal antibody that blocks the activation of tyrosine kinase –dependent molecular signaling pathway by binding to extracellular domain of epidermal growth factor receptor (EGFR) was approved by FDA for the treatment of locoregionally-advanced (LA), as well as recurrent/metastatic disease [93]. Overexpression of EGFR has been detected in majority of HNC patients (approximately 90%), and is in the same time correlated to decreased survival rate and RT resistance [94]. Clinical studies have demonstrated significant improvement in OS with cetuximab addition to the EXTREME regimen (cisplatin or carboplatin, and 5-FU), thus becoming a standard of care for R/M HNC patients in the past decade [93,95]. So far, cetuximab is the only FDA approved anti-EGFR antibody for the treatment of HNC patients [96]. 

Similarly, taking into consideration good prognosis of HPV-negative OPSCC patients with <10 pack-year smoking history, long-term toxicity of patients was evaluated in a clinical trial (NCT01663259) where concurrent CT was replaced with cetuximab. The patients were treated with concurrent RT of 70 Gy in 35 fractions together with cetuximab. Following treatment regimen, the study results have indicated two-year RR, PFS, and OS of 19%, 81%, 95.2%, respectively, with two-year freedom from loco-regional progression (LRP) of 87.9%. Cetuximab in combination with RT has resulted in mild to moderate adverse events (AE) in 33.3% of the patients, while 66.7% of the patients have developed severe AE. On the contrary to favorable therapeutic outcomes reflecting OS, PFS, still AE were observed in patients, with all grade toxicities including oral mucositis (100%), dysphagia (88.1%), and hematologic toxicity (31%). Several on-going clinical trials (De-ESCALaTE, NCT01874171, TROG 12.01 NCT01855451) including treatment with RT and cetuximab are evaluating optimum treatment strategies for HPV-negative OPSCC patients in order to decrease the toxicity rate and improve patient QoL. 

Besides cetuximab and other monoclonal antibodies (mAbs) targeting EGFR in HNSCC patients, immunotherapy in the treatment of HNSCC has been recognized as a potential approach for improvement of clinical outcomes in patients [92]. In immunotherapy, host immune system cells recognize cancer cells as foreign, triggering destruction mechanisms to eliminate the target cells [97]. Importantly, cancer cells activate different mechanism to promote self-survival, including immune escape, as well as T-cell exhaustion [98]. One of the key mechanisms of cancer immune escape is the activation of programmed death-1 or PD-1/PD-L1 signaling pathway [65,89]. Overexpression of PD-1 has been observed on cluster domain 8 (CD8+) tumor-infiltrating lymphocytes in HPV-positive patients and it is in the same time linked with better five-year OS compared to HNSCC patients with low PD-1 expression (93.9% vs. 63.6%), respectively [97,99]. Except PD-1 checkpoint, cytotoxic T-lymphocyte-associated protein 4 (CTLA-4) is also potential target for HNSCC immunotherapy [98]. Thus, inhibition of PD-1/PD-L1 signaling with anti-PD1/PD-L1 agents in order to inhibit immunosuppressive response of tumor cells represents a promising approach in the treatment of HNSCC patients [65]. So far, two anti-PD-1 antibodies, nivolumab and pembrolizumab are approved by FDA for the treatment of R/M HNSCC patients. However, response rates to immunotherapeutic agents in HNSCC patients range from 13–20%, with significantly improved OS rate in one out of ten patients [65,92]. Nivolumab and pembrolizumab monoclonal anti-PD-1 antibodies have been investigated through clinical studies in the treatment of patients with HNSCC [70]. The effects of nivolumab were investigated in a randomized phase III clinical trial (CheckMate 141, NCT02105636) for the treatment of platinum-refractory HNSCC [98]. The results have shown OS rate of 7.49 months compared to the standard of care treatment arm receiving cetuximab, methotrexate or docetaxel, where OS of patients was 5.06 months. Based on clinical outcomes, nivolumab obtained FDA approval in 2016 as the first anti-PD1 mAb for the treatment of R/M HNSCC refractory to platinum-based therapy [86].

In an open-label, phase 1b clinical trial (KEYNOTE-012), safety, tolerability and anti-cancer effects of pembrolizumab in patients with PD-1+ R/M HNSCC were evaluated (NCT01848834). From the total number of PD-1+ patients (*n* = 60), 38% were HPV-positive patients. OR by central imaging review was achieved in 18% of all patients, with greater response in HPV-positive patients. Additionally, pembrolizumab treatment was well-tolerated, where 17% of the cohort had stage 3–4 drug related AE, and 45% of the patients experienced serious adverse events (SAE). Showing significant clinical outcome in R/M HNSCC patients with high PD-1 expression, the effects of pembrolizumab on R/M HNSCC was evaluated in a follow-up, phase III KEYNOTE-040 clinical trial (NCT02252042) [87]. The patients were divided into two treatment groups, including pembrolizumab treatment, as well as comparator group receiving methotrexate, docetaxel or cetuximab. Two-year OS rate in pembrolizumab treatment group was 8.4 months, while OS in active comparator group was 6.9 months. Additionally, objective response rate (ORR) by RECIST 1.1 central imaging was 14.6 and 10.1 months, respectively. Moreover, in a randomized, phase III clinical trial (KEYNOTE-048, NCT02358031), OS in participants treated with pembrolizumab and CT in combination was 13 months, compared to control group receiving cetuximab and CT with OS of 10.7 months. Based on PD-1 combined positive score (CPS) > 1, OS rate in pembrolizumab mono-treatment arm was 13.6 months in contrast to control group receiving cetuximab and CT (10.4 months). Finally, pembrolizumab was approved by FDA in 2019 as a first-line treatment option for R/M HNSCC patients with PD-1 combined positive score > 1 (www.fda.gov; accessed on 23 March 2021). These data emphasize the exigency for evaluation of novel biomarkers in HNSCC patients with an aim of improving response and OS rates of the patients. 

In the recent years, prevention of HPV-related infections that might lead to cancer has been emphasized. Currently available vaccines Cervarix, Gardasil-4 and Gardasil-9 are approved for prevention of cervical cancer, vaginal cancer, penile cancer, and genital warts caused by HPV viruses [47]. Bivalent vaccine Cervarix is used for the prevention of HPV-16 and 18, Gardasil-4 is quadrivalent vaccines targeting HPV-16, 18, 6, and 11, while Gardasil-9 is 9-valent vaccine that, besides the aforementioned HPV subtypes, also targets HPV-31, 33, 45, 52, 58 [100]. Although recent research suggests that vaccines might be effective in the prevention of oral HPV infections, more clinical trials examining the decrease in OPSCC after vaccination are urgently required [101]. The efficacy of the Cervarix vaccine in preventing cervical and oral infections four years after vaccination was evaluated in a phase III clinical trial (NCT00128661). Among 91.9% of eligible patients (*n* = 5840), oral prevalence of HPV was detected in 1.7% of individuals, with the vaccine efficacy of 93.3% [102]. Similarly, in another study, oral HPV infection incidence in vaccinated population was decreased in comparison to unvaccinated patients [103], with prevalence of HPV infection of only 5.6%. Moreover, Chaturvedi et al. (2018) showed reduction of HPV16, 18, 6, 11 oral infection prevalence for 88.2% in vaccinated US population [36]. Finally, these data suggest significant decrease in oral HPV infections in vaccinated individuals, underlining the importance of prevention strategies for HPV-induced OPSCC [100].

## 3. Epstein-Barr Virus

Epstein–Barr virus (EBV), also known as human herpes virus type 4 (HHV4), is one of the nine identified human herpesvirus types in the herpes family, and one of the most abundant viruses in human population [41]. EBV was discovered by Epstein’s group in a Burkitt’s lymphoma cell line in 1964 using electron microscopy as the first human tumor-related virus [10,41,50]. According to the International Agency for Research on Cancer (IARC), EBV is graded as a Group I carcinogen since its relation to certain lymphoid and epithelial malignancies. Globally, many people are infected by EBV and carry the virus throughout their life, usually without any harsh symptoms. However, persistent EBV infection can lead to the development of malignancies [19]. EBV infects roughly 90–95% of all adults globally and causes approximately 1% of all cancers, including Burkitt’s lymphoma (BL), diffuse large B cell lymphoma (DLBCL), gastric carcinoma (GC), Hodgkin’s lymphoma (HL) and nasopharyngeal carcinoma (NPC) [41,104,105]. The EBV has long been correlated only to NPC development, but recent meta-analysis study showed association of EBV with OSCCs development [50].

### 3.1. EBV Genome and Mechanism of Infection

The EBV genome consists of linear double-stranded DNA, approximately 180 kb pairs long, containing 85 genes (Table 1) [10]. The EBV exhibits dual tropism, infecting both, B cells and epithelial cells, due to the ability to alternate its cell entry mechanism by switching envelop proteins. EBV is known to use the glycoprotein gp350 envelope protein for binding to the complement receptor type 2 protein present on the membrane surface of B-cells [10]. However, while infecting epithelial cells, it switches to the gp40 envelop protein and binds to the surface integrins. The proteins used in different ways of infection and cell entry mechanisms are very important trait of EBV’s perseverance in humans [10,106]. Infection of B-cells starts by binding of EBV glycoprotein gp350 to cell receptor CD21 and interaction of viral glycoprotein gp42 with MHC class II molecules. This triggers fusion of the viral envelope with the cell membrane, allowing EBV to enter the B cell [106,107]. In CD21 negative human cells, CD35, also called complement receptor 1 (CR1), represents another binding factor for gp350/220, allowing the entry of EBV [106]. In order to evade epithelial cells, viral proteins BMRF-2 and gH/gL interact with cellular β1 and αvβ6/αvβ8 integrins, respectively. This promotes fusion of the viral envelope with the epithelial cell membrane, permitting EBV entrance to the epithelial cell [106,107]. In contrast to B-cell entry, epithelial-cell entry is obstructed by viral glycoprotein gp42 [107].

Upon encountering host cells, the viral capsid dissolves and the viral genome is transported to the cell nucleus. EBV is able to undergo both latent and lytic phases of infection. In latent infection, the EBV genome replication occurs only once with each cell cycle. During lytic infection, the EBV genome is replicated to produce a high number of viral genomes, packaged in infectious particles for transmission. EBV infection in normal epithelium follows the lytic infection mode, whereas latent infection is the principal mode of EBV infection in epithelial cancers such as NPC and EBV-associated gastric carcinoma. Consequently, the latent EBV infection represents a critical hallmark of the pathogenesis of EBV-associated epithelial cancers. Most probably, some somatic mutations and changes in cell signaling in premalignant epithelial cells enhance the switch of the default lytic infection to latent infection, enabling the persistent infection of EBV in epithelial cells [41].

Proteins expressed by the EBV genome, including LMP1, LMP2, and EBNA1, are involved in maintaining the oncogenic properties of the virus and control cancer at every stage, from oncogenesis to progression and metastasis [108,109].

LMP1 has three functional domains in its *C*-terminal region, *C*-terminal activating regions 1, 2, and 3 (CTAR1, CTAR2, and CTAR3) that can activate NF-κB, JNK (c-Jun *N*-terminal kinase), p38 MAPK, JAK/STAT, and PI3K/Akt signal pathways involved in cell cycle progression [108,109]. Cells expressing LMP1 have nonfunctional G2 cell cycle checkpoint and progressive tumorigenesis mediated by the matrix metalloproteases (MMPs). MMPs help degrade extracellular matrix, making the cells vulnerable to the virus. LMP1 can regulate p53 and epidermal growth factor receptor (EGFR) resulting in promoted cell cycle progression through downregulation of the CDK inhibitor p27^Kip1^, CDK2, and Rb [109]. Additionally, LMP1 can regulate telomerase activity via the p16^INK4A^/Rb, PI3K-AKT and JNK signaling pathways to stimulate cell immortalization [110] and to regulate angiogenesis and INF-γ pathways important in oncogenic transformation of cells via mediated immune escape [108].

LMP2 viral proteins include LMP2A and LMP2B transmembrane proteins which can hinder tyrosine kinase signaling. LMP2 imitates activated B-cell receptor (BCR), obstructing regular B-cell signal transduction and can associate with Src family protein tyrosine kinases (PTKs) as well as spleen tyrosine kinase (Syk), both related with BCR signal transduction [111]. LMP-2A can influence transformation of epithelial cells through activation of the PI3-kinase–Akt pathway [112]. Like LMP1, LMP2 can also control INF-γ signaling to assure immune escape in cancer. EBNA1 protein is mostly expressed in dividing memory B cells, but due to its preservation of the viral genome in latent infections, EBNA1 is found all EBV-associated cancers including NPSCC. It assists replication of the viral episomes and promotes the survival of cells with damaged DNA [109,113]. EBNA1 can modulate pathways such as invasion, cell proliferation, survival, and DNA damage repair [109]. LMP1, LMP2, and EBNA1proteins represent relevant targets for therapeutic research in last decades, particularly in the field of immunotherapy [44].

### 3.2. EBV in HNSCC Development

EBV infection is reported in 100% of nonkeratinizing nasopharyngeal carcinomas (NPCs) [41]. Clinically, nasopharyngeal carcinoma (NPC) is exceedingly invasive and metastatic cancer type that is widely predominant in southern China [49,114]. Epidemiology, pathological types, and treatment options of NPC differs from other cancers arising from head and neck [115]. Patients diagnosed with early clinical stage disease have circa 90% OS rate. However, most patients are diagnosed in advanced stages, and the survival rate is decreased to <50% [57]. 

According to the numerous data, EBV is closely linked to NPC. Zheng et al. detected EBV genomes in almost 100% of tumor lesions in undifferentiated type NPC, squamous cell and non–keratinizing NPC in endemic areas [57]. However, low levels of EBV DNA load were also detected in some NP brushing samples from normal NP, which contradicts to the hypothesis that healthy NP epithelial cells are negative for EBV infection. Zheng et al. detected the EBV DNA load in 87.8% of NP brushing samples (*n* = 82) from the control group in the high-risk area [57].

Extensive efforts were made to find a method to simplify NPC diagnosis and screening by testing EBV-related biomarkers. Since 1990s, detecting EBV DNA load in plasma or serum was established as a strong biomarker of NPC [57]. The blood plasma EBV DNA is detected in the tumor cells of nearly all anaplastic nasopharyngeal cancers (NPCs) [49]. This method is considered the most precise molecular predictive biomarker in diagnosis and treatment [49,57,115]. Additionally, nasopharyngeal (NP) brushing/swab samples can also be used for qualitative and quantitative detection of EBV DNA. In addition, tumor makers mRNA,18 microRNA22 and tumor suppressor gene methylation can be evaluated by NP brush sampling [57].

### 3.3. Current Treatment Options and Update on Clinical Trials

EBV-positive NPC is recognized as one of the most aggressive and metastatic head and neck cancers, with a cure rate of 90% in early I–II stages [116]. As the majority of patients are asymptomatic, diagnosis usually occurs in advanced III-IV EBV-positive NPC stages, resulting in five-year survival rate of 50–60% [116,117]. Several characteristics of nonkeratinizing EBV-positive NPC exist, including high expression of PD-L1 [118], dense lymphocytic infiltration [119] and prevalence of specific genetic alterations (CCDN1, PI3K/Akt, NF-kB, and epigenetic deregulations), providing potentially novel targets for immunotherapeutic and personalized medicine approaches [117]. However, current treatment options are cancer stage-dependent: I-II NPC stage patients are treated with RT and concurrent CT, while advance stage patients are treated with CT [120]. Although first line treatment options including cisplatin and gemcitabine have resulted in high response rates, PFS remains at just seven months [121]. No drug for EBV-positive NPC has been approved to date.

The expression of EBV-specific proteins represents a promising approach for treatment strategies with checkpoint inhibitors, potentially resulting in favorable tolerance and clinical outcome [119,122]. The effects of nivolumab, an anti-PD1 antibody, were evaluated in a clinical trial with R/M NPC patients (NCT02339558) (Table 3). Following nivolumab treatment, the results indicated ORR of 20.5% and a disease control rate of 54.5%. Moreover, one-year OS and PFS among the patients were 59% and 19.3%, respectively. In regard to PD-1 expression, 33% of PD-L1-positive NPC patients responded to nivolumab treatment, compared to PD-L1-negative NPC patients, where response did not exceed 13%. In addition, patients with loss of human leukocyte antigen A (HLA-A) and/or HLA-B expression showed statistical significance in one-year PFS (30.9%) compared to patients with HLA-A/HLA-B tumor expression (5.6%). Considering promising outcomes of nivolumab treatment in pretreated NPC patients, its activity and correlation to biomarker expression should be further evaluated [117]. Therefore, anti-tumor activity of nivolumab and ipilimumab, an anti-CTLA-4 antibody as combinatorial therapy is currently under evaluation in phase II clinical trial in patients with rare tumor types, including NPC (NCT02834013) (Table 3). Importantly, the efficacy of nivolumab and other immunotherapeutic candidates is currently under phase II clinical investigation (CheckMate358, NCT02488759) in patients with virus-associated cancer, including EBV-positive NPC (Table 3). Activity of another anti-PD1 monoclonal antibody, pembrolizumab has been evaluated in multi-cohort, phase Ib clinical trial (KEYNOTE-028, NCT02054806) in patients with R/M NPC expressing PD-L1 (93.2%) (Table 3). In this study, ORR by investigator review was achieved in 25.9% of the patients, with one-year PFS rate of 33.4%. Conclusively, pembrolizumab has demonstrated favorable anti-cancer activity and acceptable safety profile in NPC patients [123]. 

In addition to immunotherapy, the adoptive transfer of cytotoxic T-lymphocytes (CTLs) specific to EBV that target LMP1, LMP2, and EBNA1 viral antigens has been recognized as potential approach for endemic NPC patients [44]. In order to improve the patient’s clinical outcome, CT including gemcitabine and carboplatin (four cycles) was administered to NPC patients (*n* = 35), following EBV-CTL (six doses). Therapeutic response rate was 71.4%, with two-year OS of 62.9% and three-year OS of 37.1%. Thus, addition of CTL with T-cells specific for EBV LMP2 were significantly correlated to OS rates. Interestingly, after adoptive CTL transfer, 14.3% of the patients did not require further CT treatment for 34 months. As a result of outstanding OS rates in locally recurrent or metastatic EBV-positive NPC patients [124], currently ongoing phase III clinical trial (NCT02578641) will evaluate therapeutic outcome of carboplatin and gemcitabine with or without EBV CTL therapy in patients with metastatic EBV-positive NPC. CTLs have drawn a great attention in the treatment of EBV-positive NPC. Multiple clinical trials are evaluating the efficacy of specific CTLs, including LMP1 and LMP2 (NCT00516087), CTL treatment with prior treatment with monoclonal CD45 antibody (NCT00706316), however, the results are not available yet (Table 3). Multiple treatment strategies for NPC have been investigated through clinical trials, including EGFR inhibitors, PI3K/Akt inhibitors, inhibitors of angiogenesis, and other, however no drug has been approved for the treatment of NPC. However, EBV-positive NPC has a specific molecular signature where immunotherapy represents a promising approach in EBV-positive NPC management, especially adoptive EBV CTL that are under clinical investigation [44,117].

Furthermore, high expression of EBV-specific proteins, such as LMP1, LMP2, and most importantly, EBNA1, represents a promising target for the vaccine development [10]. Among the crucial factors for EBNA1 as an immunotherapeutic target are its involvement in the regulation of molecular signaling, maintenance of EBV DNA, and the presence of multiple CD4+ T-cell epitopes. Immunological competence of LMP2 is observed through its expression of large number of CD8+ T-cell epitopes [10,125].

**Table 3:** Clinical trials in EBV-related nasopharengyal cancer.

Clinical Trial/NCT Number/Year	Phase	Disease Stage	Patient Number (*n*)	Treatment Arms	Outcome
NCI-9742 NCT02339558 2015–2019 [119]	Phase II	Nonkeratinizing, R/M NPC, stage III-IVc	*n* = 45; Plasma EBV DNA detection (*n* = 44)	Nivolumab (3 mg/kg for 4 weeks)	ORR: 20.5% one-year outcome: OS: 59% PFS:19.3%
KEYNOTE-028 NCT02054806 2014–2020 [123]	Phase I	PD-L1-positive, R/M NPC	*n* = 27	Pembrolizumab (10 mg/kg every 2-week cycle for 24 months)	ORR: 25.9%; one-year PFS: 33.4%
Adoptive T-cell transfer NCT02578641 2008–2011 [124]	Phase II	EBV-positive R/M NPC	*n* = 38	Venesection → CT (gemcitabine 1000 mg/m^2^ and carboplatin AUC 2 every 4 weeks for 4 cycles) + EBV-CTLs ^1^ (1 × 10^8^ cells/m^2^ on weeks 0, 2, 8, 16, 24, 32)	RR: 71.4%; Median OS: 29.9 months; two- and three-year OS: 62.9% vs. 37.1%
MVA-EBNA1/LMP2 (MVA-EL) NCT01256853 2006–2010 [126]	Phase I	EBV-positive NPC	*n* = 18	MVA-EL vaccine (3 intradermal vaccinations at 3-week period, with doses of 5 × 10^7^, 1 × 10^8^, 2 × 10^8^, 3.3 × 10^8^, 5 × 10^8^ plaque forming units (pfu)	T-cell response (one or both antigens): 15 patients
MVA-EL NCT01147991 2005–2010 [125]	Phase Ia	EBV-positive NPC	*n* = 16	MVA-EL vaccine (3 intradermal vaccinations at 3-week period, doses of 5 × 10^7^–5 × 10^8^ pfu)	T-cell response (one or both antigens): 8 patients (7/14, EBNA1; 6/14 LMP2)

^1^ CTL—cytotoxic T-lymphocytes.

The first clinical trials evaluating vaccination strategies for NPC used LMP2 CD8+ T-cell epitope peptides loaded into autologous monocyte-derived dendritic cells. Out of 16 patients, LMP2 T-cells were detected in 9, with partial CR in 2 patients [127]. The presence of the abovementioned EBV targets has drawn interest in the implementation of immunotherapeutic strategies in the treatment of NPC. Unfortunately, vaccination with autologous monocyte-derived DCs is limited to specialized centers with trained personnel, thus excluding treatment on a large scale [10]. Therefore, Hui and colleagues (2013) presented a distinct immunotherapeutic approach based on vaccination with recombinant vectors, known as Modified Vaccinia Ankara (MVA). MVA virus encodes inactivated fusion protein with half of EBNA1 *C*-terminal coding for epitopes but lacks specific repeat that results in failure in sequence presentation to CD8+ T-cells. Additionally, the MVA virus also encodes LMP2A [126]. In phase I clinical study (NCT01256853), patients in remission having EBV-positive NPC (*n* = 18) were treated with three MVA-EL vaccines at escalating doses. Out of eighteen patients, T-cell response mapped to certain CD4 and CD8 EBNA1 and LMP2 epitopes was observed in fifteen patients. Importantly, strong responses were observed in patients treated with the highest dose, suggesting a good safety profile and immunogenicity of MVA-EL. Considering its safe profile and favorable treatment outcome, the safety and immunogenicity of MVA-EL was evaluated in a follow-up phase I clinical trial (NCT01147991) among fourteen patients in remission with EBV-positive NPC. The results revealed good tolerance of the vaccine, as well as the development of immunogenicity to at least one antigen in eight patients [128]. Based on promising results, the clinical benefit rate of the MVA-EL vaccine is currently under evaluation in a phase II clinical trial with R/M NPC patients with residual EBV DNA load following conventional treatment regimen (NCT01094405) (Table 3) [10].

## 4. Hepatitis C Virus

Hepatitis C virus (HCV), a member of the Flaviviridae family, is an important public health problem worldwide [51]. The overall HCV incidence is estimated to be 2%, with nearly 180 million infected people and the great majority with chronic HCV infection [51]. These oncogenic pathogens are associated with the hepatocellular carcinoma and with smoking and alcohol-related cancers, including cancers of the pancreas, lung, and kidney, and non-Hodgkin lymphoma [42,51,52]. Recently, many studies have provided evidence that a large number of patients diagnosed with HNSCC also have HCV [42,51,52,129,130,131]. The overall HCV prevalence in patients diagnosed with HNC was 7.8%, reaching 12.8% in HNSCC and 3.4% in other head and neck malignancies [131].

### 4.1. HCV Genome and Mechanism of Infection

HCV is a single stranded RNA virus whose genome has single open reading frame (ORF) 9600 bp long (Table 1) [37]. The *N*-terminal part of the ORF encodes structural proteins including core and envelope glycoproteins, whereas the remaining part of the ORF encodes for the nonstructural proteins [40]. 

HCV has triple tropism and can infect B-cells, liver cells and salivary gland cells, and is transmitted utterly through direct blood-to-blood contacts between humans. [42]. Once it infects the host cell, HCV can trigger a range of pro-malignant mechanisms, starting with ability to evade host immune response which targets HCV by triggering cell cycle arrest and apoptosis. The mechanism of HCV infection associated with HNCs is still not fully elucidated and merits further investigation. Oral lichen planus, an extrahepatic manifestation of chronic HCV infection, is a premalignant form of squamous cell carcinoma which develops in the oral cavity, as reported by case studies [132,133]. Increased HCV replication rates in OPSCC tissues possibly cause chronic inflammation as a predisposition to cancer development [45,58]. Carcinogenesis of HCV in HNC closely depends on HCV proteins, including core and nonstructural protein 3 (NS3) or NS5A [51]. Like the HPV E6 and E7 oncoproteins, HCV proteins also disrupt cell cycle regulation by proteasomal degradation of Rb and loss of the p53 function [45,51]. Also, HCV proteins are able to diminish the activity of the transforming growth factor β (TGF-β), whose function is to inhibit growth by inducing apoptosis in epithelial cells [51].

### 4.2. HCV and HNSCC Development

Association studies have reported increased risk of HNSCC development in HCV infected patients, but those conclusions are usually limited by the small sample sizes. Therefore, Brosetto et al. (2020) performed a meta-analysis to summarize present reports about HCV infection in HNSCC. Upon the literature search, they included eight observational studies investigating the relationship between HCV and cancers of oral cavity, oropharynx, hypopharynx or larynx [51]. Their results proposed a significant effect of HCV in both oral (RR = 2.13; 95%: 1.61–2.83), oropharyngeal (RR = 1.81; 95% CI: 1.21–2.72), and laryngeal HNSCC (RR = 2.57; 95% CI: 1.11–5.94) development [51]. HCV infection has been identified in approximately 10% of HNSCC patients, proposing its role in the HNC etiology [51]. This percentage is enormously heterogeneous across the geographic area, from 3% prevalence in Italy [52] to more than 20% in USA and Japan [53].

Rangel et al. (2018) reported a higher prevalence of HCV infection among patients with HNC compared to the general population in Brazil [131]. Even though the HCV infection was associated with an increased risk of HNC, the infection did not worsen the HNC scenario [131]. Moreover, a retrospective cohort study in India showed that out of 19,137 seropositive HNSCC patients, 156 patients had HBV or HCV infection, among which, HBV infection was present in majority (*n* = 86/156, 55.1%) followed by and HCV infection (*n* = 29/156, 18.6%) [54]. Most commonly, cancer developed in the oral cavity and the three-year OS for patients with coexisting HBV, and HCV infection was 62.6%, and 57.5%, respectively [54]. Similarly, Sue et al. (2012), reported the incidence of oral cavity cancers as 2.28-fold greater among patients with HCV alone than nonviral hepatitis group in Southeast Asia population [42]. The association was highest among 40–50-year old patients [42]. However, HNSCC patients are often diagnosed in the advanced stage but have a good OS with appropriate treatment [54]. A study from United States with 409 case subjects (164 OPSCC and 245 nonoropharyngeal) and 694 control subjects (378 lung, 168 esophagus, and 148 urinary bladder) showed that prevalence of HCV positive samples was higher in OPSCC and nonoropharyngeal cancer patients compared to control subjects [45]. Another finding of this study was HCV association with laryngeal squamous cell carcinomas (LSCC). Risk factors for LSCC are smoking, alcohol and drug use, gastroesophageal reflux, and job-related contact with asbestos. However, the association of HCV with LSCC may be due to the mechanisms similar in HPV-positive cases. Correlation between smoking and/or drug use could partially explain the association of HCV with laryngeal cancers [45]. 

HCV infection diagnosis has advanced from serologic detection of nonspecific anti-HCV antibodies to targeting viral RNA in serum by the polymerase chain reaction (PCR) method [134,135]. This offers fast, accurate and economic detection, which could also distinguish present from past infection, offering better understanding of HCV-related HNC development [135]. With the worldwide high prevalence of HCV, hepatologists should be encouraged to additionally observe patients with known chronic HCV infections to assist early HNSCC diagnosis. Further studies are necessary to clarify the direct role of HCV replication and the indirect role of HCV proteins in HNSCC development [51].

## 5. Hepatitis B Virus

Hepatitis B virus (HBV) afflicts more than 300 million people worldwide, resulting in nearly one million deaths annually [39,55]. Approximately 75% infected people live in the Asia Pacific region, predominantly in Southern China [55]. HBV belongs to the *Hepadnaviridae* family, and has been known to infect humans since the Bronze Age [38]. Like HCV, HBV is a carcinogenic pathogen and is associated with hepatocellular and pancreatic carcinoma and non-Hodgkin’s lymphoma [42,52].

### 5.1. HBV Genome and Mechanism of Infection

HBV is a small enveloped virus with a circular double-stranded DNA genome of approximate 3.2 kb (Table 1) [38,39]. The viral genome encodes four overlapping genes called S, C, P and X. The S gene encodes the viral envelope surface antigens (HBsAgs) proteins, whereas the C gene encodes the 21-kDa core protein and the 25-kDa pre-core protein. The core protein contains the pre-genomic RNA (pgRNA), usually encapsulated by the HBV polymerase [39,46]. The P gene encodes the viral DNA polymerase acting as a reverse transcriptase, and the X gene encodes for a protein with multiple functions required for viral replication [46].

HBV has dual tropism, infecting both hepatic cells and B-cells. Additionally, it can replicate in lymphocytes and peripheral blood mononuclear cells [43] (Table 1). Upon infection of the host cell, the HBV genome is delivered into the nucleus and forms a covalently closed circular DNA (cccDNA), which functions as the template to direct transcription of viral RNA [38,39]. The cccDNA is exceedingly stable in the nucleus of infected host cell, making the chronic HBV infection difficult to treat and allowing the virus reappearance after the treatment termination. Upon formation of the viral core particle, the HBV pgRNA is used as a template to synthesize single-stranded linear and then partially double-stranded circular DNA. Viral particles containing DNA genome are then released from infected host cells [38,39,46]. HBV is not a cytolytic virus and due to this, many HBV carriers are asymptomatic and virus can persist in infected cells [38,39].

Since HBV is known not to infect epithelial cells, data explaining a potential link between HBV infection and nonliver cancer is limited. In contrast to HCV, the mechanisms of HBV carcinogenesis are still not clear. However, HBV possibly exerts both direct and indirect oncogenic effects on cancer development [58]. Direct effects include both p53 protein suppression by HBV X protein and viral genome integration [136]. Indirect effects depend on hepatocyte death and regeneration cycle related to chronic hepatitis, which predispose the patient to malignancy [137]. The inflammatory mechanism in which hepatitis causes liver cirrhosis and cancer is clear. However, a mechanism of hepatocellular carcinoma development from a latent hepatitis virus infection may suggest the existence of a different carcinogenic routes. It is likely that HBV infection promotes carcinogenesis in HNC by one or more of these mechanisms [58], but few studies on this topic are currently available.

### 5.2. HBV and HNSCC Development

Su et al. reported a study where infection with HBV alone and HBV/HCV coinfection were not associated with oral cavity cancer incidence [42]. In line with this, a study reported in the Japanese population showed that elevated HBV surface antigen levels were present in patients with benign oral tumors, but not in those with oral cavity cancer which needed dental surgery [138], suggesting that HBV infection does not stimulate oral tumor formation [42]. In contrast, in a Southeast Asian population, where the prevalence of HBV is up to 2.7%, HBV was associated with oral cancer [54].

A few studies have shown that Southern China has a higher NPC incidence compared to other regions [43,55]. Also, HBV infection is endemic in this region, suggesting that a considerable percentage of NPC patients have HBV infection. The first study to report association of HBV infection with an increased risk of NPC development in Southern China was a large-scale case-control study performed by Ye et al. in 2015 [55]. 

HBV infection represents an indirect risk factor in the etiology of NPC, since it reduces immune functions compared to noninfected group [43,55]. However, it is assumed that HBV interacts with EBV and stimulate the NPC pathogenesis. Typically, EBV is present in B-cells in latent phase under the strict watch of the host immune system. However, HBV infection can trigger B-cells, potentially activating or reactivating the latent EBV which increase the EBV infection of epithelial cells [55].

Weng et al. have concluded that HBV treatment can increase prognosis chances of HBV-infected NPC patients [43]. Out of 876 patients, 106 (12.1%) were positive for HBV infection, without any major differences in gender and cancer stage, but the HBV positive group mostly included patients age above 50 years compared to the HBV negative group [43].

### 5.3. Treatment Options for Hepatitis C and B Viruses

HBV is found in approximately 11% of NPC patients, suggesting a correlation of HBV infection in NPC patients [43]. Patients characterized with resolved HBV infection and low serum levels of HBV replication are found to be prone to infection reactivation as a result of immunosuppressive effect of CT [139]. Unfortunately, these complications might induce liver damage and lead to poorer prognosis. Considering issues that might occur posterior to the treatment, US Centers for Disease Control and Prevention suggested HBV screening prior to exposure to cytotoxic drugs [139]. Yeo et al. (2005) evaluated the incidence of HBV reactivation with prophylactic lamivudine in NPC patients undergoing CT [140]. Compared to the control group treated with CT only (*n* = 21), the study group treated with lamivudine (*n* = 16) before and after CT has resulted in decreased incidence of hepatitis (33.3% vs. 6.7%), lower CT disruption (67.7% vs. 18.8%), with 28.6% of HBV reactivation in control group compared to subjects treated with antiretroviral lamivudine drug (0%). Without CT disruption, these data might indicate more favorable survival rates of HBsAg-positive NPC patients with prophylactic lamivudine treatment [140]. Weng and colleagues have shown that prognosis of early-stage NPC patients is correlated to HBV infection, where antiviral treatment with entecavir, lamivudine, and/or adefovir before and after CT has improved DMFS compared to the patients who did not undergo antiviral therapy (90.0% vs. 70.0%, respectively). These data underline the importance of anti-viral treatment in order to improve prognosis in HBV-positive NPC patients [43]. Moreover, Xu et al. (2015) have evaluated the clinical characteristics and prognosis in newly diagnosed NPC patients with confirmed HBV infection (*n* = 722). After receiving comprehensive treatment, the patients with HBsAg (+) had a worse clinical outcome, with more common distant failure rate compared to HBsAh (−) group (31.6% vs. 12.9%, respectively). In addition, positive carriers had a poorer five-year DFS (69.2% vs. 86.8%), PFS (58.5% vs. 77.4%), and OS rate (66.6% vs. 79.0%) in comparison to negative carriers [141]. Similarly, Nayyar et al. (2020) evaluated the oncologic outcome of human immunodeficiency virus (HIV), HBV and/or HCV seropositive HNSCC patients treated with standard of care regimen for specific site and disease stage. Following curative treatment, DSS in HBV and HCV patients was 78.6% and 53.8%, respectively, with three-year OS rate of 62.6% for HBV and 57.5% for HCV-infected patients. However, as the results indicate similar survival rates among seropositive and nonseropositive patients, the general prognosis should remain the same for both patient groups [54]. 

Furthermore, a significant association of HCV infection and incidence of various malignancies was reported in recent decades, including HNC. In one study, patients with oropharyngeal (*n* = 164) and nonoropharyngeal cancer including oral cavity, nasopharynx, hypopharynx, and larynx (*n* = 245) were included. The results showed significant prevalence and association of HCV with HPV-associated oropharyngeal and nonoropharyngeal (except nasopharyngeal) cancer patients compared to control group. Possible association of HCV and HPV should be elucidated in further studies [45]. Moreover, Su et al. (2019) conducted a study to evaluate the effect of pegylated interferon and ribavirin antiviral therapy on the oral cancer development. The results indicated significantly decreased risk for development of oral cancer after undertaking antiviral therapy (35% decreased risk). Also, patients enrolled in the study had a greater risk of oral cancer if characterized with HCV infection [42].

## 6. Merkel Cell Polyomavirus

Merkel cell polyomavirus (MCPyV) was also acknowledged as an etiological factor in HNC development [142]. MCPyV belongs to the Polyomaviridae virus family and is a nonenveloped dsDNA virus implicated in Merkel cell carcinoma (MCC), a rare aggressive neuroendocrine skin cancer [143,144]. Other members of polyomaviridae family include BKPyV, JCPyV, HPyV6, HPyV7, HPyV9, HPyV10, HPyV12, KIPyV, LIPyV, WUPyV, NJPyV, STLPyV, and TSPyV [143]. The oncogenic role of MCPyV BKPyV and JCPyV polyomaviruses remains controversial [145]. However, the prevalence of MCPyV has been detected in OSCC, pharyngeal cancer, and other HNSCC types [142]. Infection with human polyomavirus occurs in childhood, at which time it is asymptomatic, and remains for decades in infected individuals after initial contact [146]. Even though the virus can be found on skin of healthy individuals, it is mainly associated with immunocompromised and immunosuppressed patients [143].

The polyomaviruses genome is composed of three parts, where early gene region encodes small T antigen and large T antigen independently involved in cancer cell proliferation and survival [146]. The large T antigen deregulates the cell cycle and apoptosis by targeting pRB, p107, and p130, resulting in downstream survivin protein activation as an important factor for the proliferation of cancer cells. In contrast, major transforming oncogene and small T antigen activate 4E-BP1 translation regulator, leading to cell proliferation [142,145,146]. T antigens also enhance c-myc promoter transcription by interaction with beta-catenin [145]. The other two functional genome parts include the noncoding control region with promoter elements and transcription start site, while the late region of the viral genome encodes three capsid proteins, VP-1, -2, and -3 [146].

The role and prevalence of human polyomaviruses in HNSCC has not been clearly established so far, and is currently under investigation in a number of studies. Muñoz and colleagues (2020) showed significant association of MCPyV with HNSCC in Chilean patients, where MCPyVs were detected in 12.5% of the cohort (*n* = 120 in total). However, BKPyV was detected in 0.8% of the patients (*n* = 1), while there were no reported JCPyV infections in HNSCC cohort involved in the study [145]. Furthermore, the impact of MCPyV on development of HNSCC was evaluated among Iranian patients. In this study, 50 HNSCC biopsy samples, including the same number of noncancerous adjacent tissues were taken from histopathologic sites of tongue, throat, lip, cheek, and submandibular region of the patients. DNA MCPyV was detected in 16% of the patients (*n* = 8), with strong association of HNSCC stages and MCPyV DNA load present in the highest extent in stage III HNSCC. However, further studies are warranted to understand the potential role of MCPyV in development of HNSCC [143]. Moreover, in a pilot study conducted by Hamiter and colleagues (2017), MCPyV DNA was detected in 28.6% of the patients with SCC of the tongue (*n* = 6/21), while all biopsy samples taken from normal base of tongue in noncancerous individuals were negative for MCPyV DNA. In another study, the presence of MCPyV and other polyomaviruses, including HPyV6 and HPyV7 were evaluated in nonmalignant tonsils (*n* = 108) and tonsilar squamous cell carcinomas (*n* = 112). The results have shown increased prevalence of MCPyV DNA in cancer compared to nonmalignant tissues (35.7% vs. 10.2%), while the prevalence of other two polyomaviruses was identical in tumor and noncancerous tissues [147].

In contrast to the abovementioned studies, Mulder et al. (2021) did not detect MCPyV among HNSCC patients (*n* = 119) without smoking and/or drinking history by IHC analysis against large T antigen expression, while HPV and EBV were detected in OPSCC and NPSCC, respectively [142]. Similarly, Windon et al. (2020) evaluated the presence of MCPyV in oral cavity cancer patients (*n* = 126). Out of the 44 cases tested for large T antigen expression by IHC, none of the tumors expressed MCPyV, thus excluding MCPyV as an etiologic factor for development of oral cavity cancer [148]. In another study, DNA prevalence and load of different HPyVs were evaluated in tonsillar squamous cell carcinoma (*n* = 38) and noncancerous tonsillar tissue (*n* = 40). MCPyV and HPyV6 were detected in a higher ratio in cancer versus nonmalignant tissue, however the differences remain nonsignificant [149]. In conclusion, the role of MCPyV and other human polyomaviruses in induction of HNSCC remains controversial. Further studies are warranted to closely understand etiology and clinical consequences of MCPyV and other viruses in HNSCC development [145].

## 7. Conclusions

Head and neck cancers include a broad spectrum of tumors which are major health problems worldwide. Risk factors for HNC development include long-term, excessive tobacco and alcohol consumption, and pathogenic infection with oncogenic viruses such as high-risk HPVs, EBV, HCV, HBV, among others.

It is important to appreciate the vastness of the range of head and neck cancers, due to the complex anatomical areas involved in their pathogenesis. Even though distinctive anatomical regions are closely located, they develop different cancers with different diagnoses and outcomes. Thus, the correct categorization of HNC is of the utmost importance. Understanding the molecular and genetic machineries which are fundamental to the carcinogenic processes and oncogenic infections is indispensable for the development of improved diagnostic tools and more effective therapies, including targeted therapies and immunotherapy. In addition, the development of therapeutic vaccines for the prevention and clearance of HPV and EBV infections offers a promising strategy for the management of HNC in the future.

## Data Availability

Can be excluded, the current paper is review on available literature.
